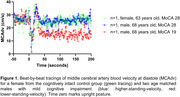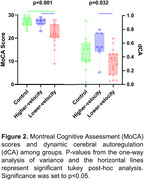# Dynamic cerebral autoregulation in cognitively intact controls and in people with mild cognitive impairment

**DOI:** 10.1002/alz70856_100441

**Published:** 2025-12-25

**Authors:** Laura Keiko Fitzgibbon‐Collins, Michael J Borrie, J Kevin Shoemaker, Jaspreet Bhangu

**Affiliations:** ^1^ Western University, London, ON, Canada; ^2^ Parkwood Institute, London, ON, Canada; ^3^ University of Western Ontario, London, ON, Canada; ^4^ St. Joseph's Health Care London, London, ON, Canada

## Abstract

**Background:**

Reductions in cerebral blood flow are associated with Alzheimer's Disease pathological changes and represent a potential therapeutic target. Measuring changes in middle cerebral artery velocity (MCAv) using transcranial Doppler ultrasound measurement of acute changes in MCAv during dynamic maneuvers and uncover relationships with cerebral autoregulation. We tested the hypothesis that changes in cerebral autoregulation are associated with clinical change in patients with mild cognitive impairment.

**Methods:**

Thirty MCI participants completed a supine‐to‐stand transition with beat‐to‐beat MCAv and mean arterial pressure (MAP) collected. Ten patients were cognitively intact and provided control measures. A 30‐second supine and standing average were calculated and a standing‐induced nadir average of the lowest 3‐beats. Dynamic cerebral autoregulation (dCA) was calculated as (MCAv_Nadir_‐MCAv_supine_/MCAv_supine_)/(MAP_Nadir_‐MAP_supine_/MAP_supine_). K‐means clustering was used to split MCI participants into higher‐standing‐velocity (*n* = 9) and lower‐standing‐velocity (*n* = 21) groups. A one‐way analysis of variance was employed to determine group differences for MoCA scores and dCA. A two‐way repeated measures ANOVA assessed group by position (supine, nadir, standing) effects for cardiorespiratory and cerebrovascular indices. Significance was set to *p* <0.05.

**Results:**

MoCA scores were significantly higher in controls and the higher‐standing‐velocity groups compared to the lower‐standing‐velocity group (Figure 2). dCA was enhanced in the lower‐standing‐velocity group compared to the higher‐standing‐velocity group (Figure 2) with an inverse relationship between dCA and standing MCAv at diastole. Interactions were observed for the resistance index, cerebrovascular resistance index, and MCAv at diastole (*p* = 0.045, 0.008, and, 0.004 respectively). Effects of position were observed for all cardiopulmonary and cerebrovascular metrics other than MCAv at systole.

**Conclusions:**

This is the first study investigating a supine‐to‐standing induced dCA response within a cohort of people with MCI. Contrary to our hypothesis, an enhanced dCA was observed in the higher‐standing‐velocity group compared to the lower‐standing‐velocity group despite the higher‐standing‐velocity group having greater cognitive scores. Interestingly, the higher‐standing‐velocity group with MCI and controls had similar MoCA scores and dCA. An enhanced dCA may be a compensatory mechanism in the neurodegenerative disease processes. The unexpected results highlight the importance of uncovering hemodynamic pathways in clinical populations and identifying the adaptions made to preserve cognitive function in the face of dementia.